# Neural plasticity in amplitude of low frequency fluctuation, cortical hub construction, regional homogeneity resulting from working memory training

**DOI:** 10.1038/s41598-017-01460-6

**Published:** 2017-05-03

**Authors:** Hikaru Takeuchi, Yasuyuki Taki, Rui Nouchi, Atsushi Sekiguchi, Yuka Kotozaki, Seishu Nakagawa, Carlos Makoto Miyauchi, Yuko Sassa, Ryuta Kawashima

**Affiliations:** 1 Division of Developmental Cognitive Neuroscience, Institute of Development, Aging and Cancer, Tohoku University, Sendai, Japan; 2 Division of Medical Neuroimaging Analysis, Department of Community Medical Supports, Tohoku Medical Megabank Organization, Tohoku University, Sendai, Japan; 3 Department of Radiology and Nuclear Medicine, Institute of Development, Aging and Cancer, Tohoku University, Sendai, Japan; 4 Human and Social Response Research Division, International Research Institute of Disaster Science, Tohoku University, Sendai, Japan; 5 Department of Functional Brain Imaging, Institute of Development, Aging and Cancer, Tohoku University, Sendai, Japan; 6 Department of Adult Mental Health, National Institute of Mental Health, National Center of Neurology and Psychiatry, Tokyo, Japan; 7 Smart Ageing International Research Center, Institute of Development, Aging and Cancer, Tohoku University, Sendai, Japan

## Abstract

Working memory training (WMT) induces changes in cognitive function and various neurological systems. Here, we investigated changes in recently developed resting state functional magnetic resonance imaging measures of global information processing [degree of the cortical hub, which may have a central role in information integration in the brain, degree centrality (DC)], the magnitude of intrinsic brain activity [fractional amplitude of low frequency fluctuation (fALFF)], and local connectivity (regional homogeneity) in young adults, who either underwent WMT or received no intervention for 4 weeks. Compared with no intervention, WMT increased DC in the anatomical cluster, including anterior cingulate cortex (ACC), to the medial prefrontal cortex (mPFC). Furthermore, WMT increased fALFF in the anatomical cluster including the right dorsolateral prefrontal cortex (DLPFC), frontopolar area and mPFC. WMT increased regional homogeneity in the anatomical cluster that spread from the precuneus to posterior cingulate cortex and posterior parietal cortex. These results suggest WMT-induced plasticity in spontaneous brain activity and global and local information processing in areas of the major networks of the brain during rest.

## Introduction

Working memory (WM) is the limited capacity storage system of the human mind and is involved in the maintenance, integration, and manipulation of information over short time periods^1^. Current knowledge of WM has been previously summarized^2^, and differences in WM capacity (WMC) underlie a wide range of higher-order cognitive functions^1^, and various neurological and psychiatric disorders^1, 3, 4^. Networks comprising the lateral prefrontal cortex (LPFC), anterior cingulate cortex (ACC), and parts of the lateral parietal lobe (e.g., inferior parietal lobule) are active during WM and play critical roles as an external attention system (EAS)^5–7^. The default mode network (DMN) is comprised of areas including the medial prefrontal cortex (mPFC), posterior cingulate cortex, and the parts of the lateral parietal lobe (mainly in the angular gyrus) is deactivated during externally directed attention demanding tasks such as WM. However, the DMN is also thought to contribute to WM performance despite the fact that DMN is deactivated during WM performance^6, 8^.

Recently, the type of working memory training (WMT) which was developed by Klingberg and colleagues^9, 10^ and characterized by the use of computer, gathered wide attention. The characteristics are summarized in the previous study as follows^11^: “*This training involves repeated performance of WM tasks, with feedback and rewards based on the accuracy for every trial*”. “*The difficulty of the tasks is adjusted during the WM training on a trial-by-trial basis by changing the amount of information to be remembered so that it is close to the capacity of the subject*.” Previous studies show that WMT can improve WM performance and inhibition/attention (for the meta-analysis, see the ref. 12). Previous studies have investigated regional changes in neurological systems through WMT, which affects white matter structure and brain activity during the task in EAS. Furthermore, WMT impacts gray matter structure involving the EAS and parts of the prefrontal cortex including the mPFC and frontopolar areas^5, 11, 13^. In addition to these studies on the effects of WMT on brain structures and task-related brain activation, our previous study and previous studies from other labs have investigated the effects of WMT on neural mechanisms during the resting state. Our previous study using MRI scans during rest revealed that WMT increases regional cerebral blood flow (rCBF) during rest^13^, increases resting state functional connectivity (RSFC) between the mPFC and the posterior cingulate cortex (increase in the RSFC between the nodes of the DMN), decreases the RSFC between the mPFC and the key nodes of the EAS, and increases the nodes of the EAS within the EAS. A study from another laboratory revealed that WMT increases the small-world nature of a distributed frontoparietal network involving the nodes of the DMN and EAS^14^.

However, the recent advancement in the methods of analyses of resting state functional magnetic resonance imaging (rsfMRI) has led to the development of new rsfMRI measures such as degree centrality, the amplitude of low-frequency fluctuation (ALFF) and fractional ALFF (fALFF; a more advanced version of the ALFF), and regional homogeneity, which are explained below. While these were used to reveal the neural mechanisms of brain diseases and disorders as well as individual differences, the effects of WMT on these three new resting state measures have not been revealed to date. The purpose of this study was to investigate this issue.

With regard to DC, recently, global brain information processing was measured in terms of the DC using rsfMRI^15^. This graph-based measurement of network organization reflects numbers of instantaneous functional connections between a given region and the rest of the brain within the entire connectivity matrix (connectome), and indicates how much of the node is used as the cortical hub^15^. The cortical hub may have a central role in integrating information across functionally segregated brain regions^16^. On the other hand, WM plays essential roles in information integration in regions around the mPFC, which is a major hub of the brain^15^, as is the frontopolar area^17^. A recent study showed that spatial WM performance is positively correlated with the mPFC, fronto-polar area, ACC, and adjacent areas as well as the posterior parts of the DMN such as the posterior cingulate cortex, precuneus, and angular gyrus^18^. However, WM performance was positively correlated with DC in the lateral prefrontal cortex, suggesting key roles of DC in this area in cognitive control^18, 19^, together with the inferior parietal lobe, which is the posterior part of the EAS^18^. Nonetheless, whether DC, functional connectomes, or spontaneous global information processing are plastic in normal adults, and how they are affected by WMT remain unknown. According to the evidence stated above, we hypothesized that WMT impacts DC in the medial to lateral prefrontal cortex and fronto-polar area as well as the parietal parts of the DMN and EAS.

With regard to fALFF, recent technical advances have enabled determinations of the ALFF and fALFF, which are suggested to indicate magnitudes of spontaneous neural activity during rest^20–22^. Consistent with this notion, repetition rates of visual stimuli induce changes in fALFF just like it does to BOLD signals to the visual cortex^23^. Furthermore, regional metabolic rates of the brain are largely accounted for by fALFF^24^. ALFF and fALFF have been used to assess neural changes in patients with clinical disorders, and have been shown to reflect different neural properties than other measurements, such as regional gray matter structure^25^. Further, ALFF and fALFF have been shown to be positively correlated with WM performance in the fronto-polar area, bilateral inferior and superior parietal lobules, and adjacent areas, which are parts of the EAS^26, 27^. However, whether and how this basic level of brain activity is affected by WMT remains unknown. Accordingly, we hypothesized that WMT impacts fALFF in the medial–lateral prefrontal cortex and fronto-polar area as well as the inferior and superior parietal lobules.

Finally, with regard to regional homogeneity, recent advances in rsfMRI analyses allow assessments of temporal homogeneity of neural activity in neighboring areas according to regional homogeneity. This local synchronicity reflects the functional homogeneity of brain areas^28^. Differences in regional homogeneity may indicate variation in the biological processes that subtend local functional connectivity^22^. While specific physiological mechanisms behind regional homogeneity are not well understood, robust data has accumulated regarding regional homogeneity in the precuneus. This region shows the highest regional homogeneity across age^29^, and reduced regional homogeneity across diseases of cognitive dysfunctions^29–32^. In particular, regional homogeneity in this area is significantly reduced with the progression of Alzheimer disease^29^, and regional homogeneity may be regarded as an index of the integrity of overall cognitive functions^33^. In addition, a previous study investigated the associations between regional homogeneity and WM performance and although this study revealed associations in a wide range of areas, the strongest positive correlation was observed in the areas of the precuneus, posterior cingulate cortex, and adjacent occipital lobe area^34^. Thus, whether regional homogeneity in this area can be increased through cognitive training is an important question. However, whether neural plasticity in regional homogeneity can be induced through cognitive training in young adults is not known. Hence, based on these previous studies, we hypothesized that WMT impacts regional homogeneity in the precuneus and the surrounding regions.

For the purpose of this study, we reanalyzed rsfMRI data from a previous report^13^ of experiments involving young adult subjects, who were went through 4-week WMT or no-intervention control before and after which subjects participated in scanning sessions that included rsfMRI (based on the data obtained in this experiment, we previously investigated the effects of WMT on RSFC between the different areas, regional gray matter structure, and rCBF during rest). Subsequently, the Data Processing Assistant for Resting-State fMRI (DPARSF) toolbox^21^, fALFF, DC, and regional homogeneity measures were applied at the whole brain in a voxel-by-voxel manner. In considering the plasticity of cognitive functions following WMT^5^, the critical roles of WM and its neural mechanisms in higher-order cognitive function^1^, changes of intrinsic brain activity, and local and global information processing in various clinical states, it is important to elucidate the extent of plasticity of global information processing (DC), intrinsic brain activity strength (fALFF), and temporal homogeneity of neural activity in neighboring areas that are assumed to reflect the overall integrity of cognitive functions.

## Material and Methods

### Subjects

**Figure 1:**
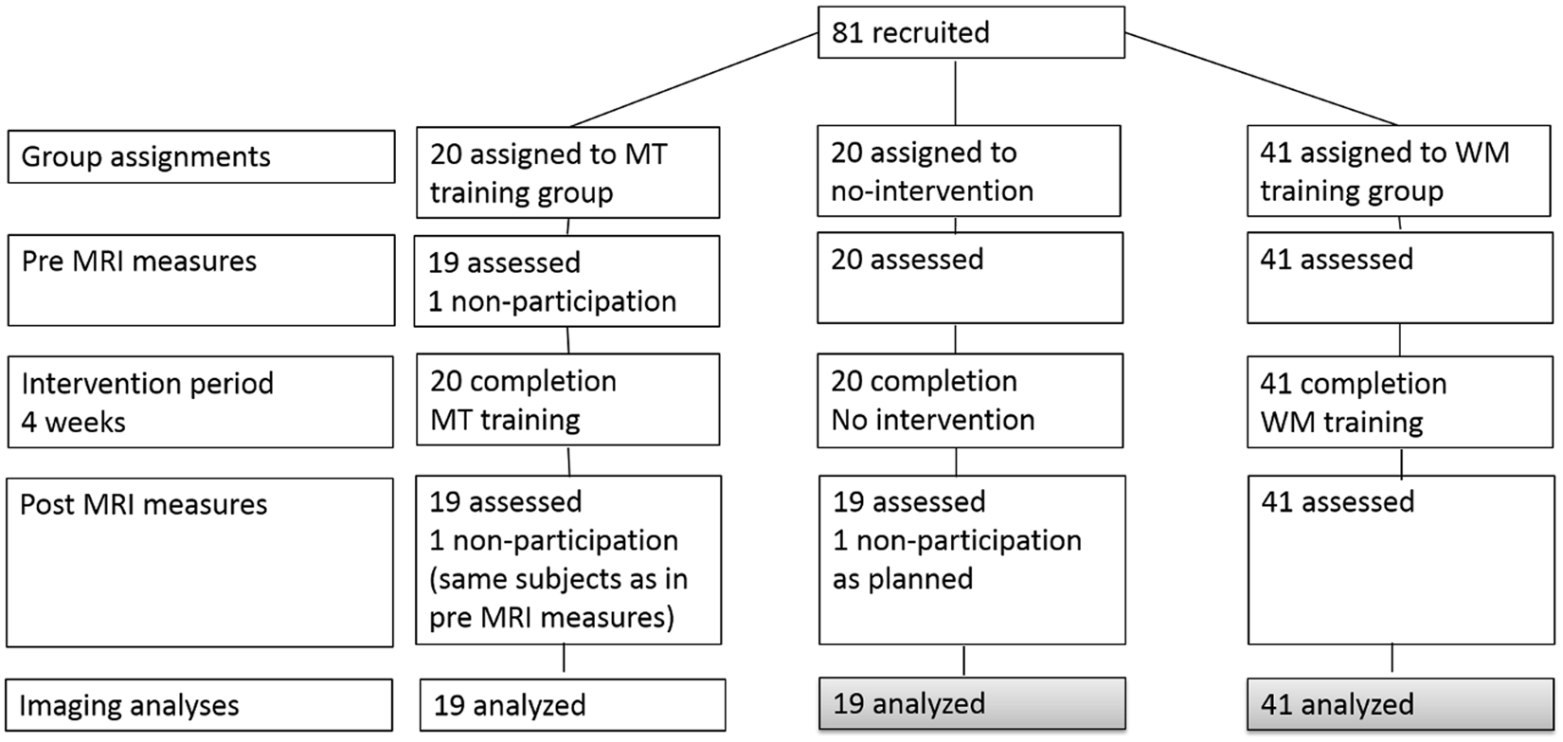
Flow of participants through the study. The data of gray parts was analyzed in this study.

As described in our previous studies^13, 35^, we enrolled 81 subjects (59 men and 22 women; mean age, 21.1 ± 1.9 years) and compared WM-training, multi-tasking training, and no-intervention control groups. Among these, the WM-training group and the no-intervention group were particularly relevant to the present study. All subjects were healthy and right-handed. The WM-training group comprised 41 participants (27 men and 14 women) with a mean age of 20.9 ± 1.6 years. The no-intervention group comprised 20 participants (15 men and 5 women) with a mean age of 21.4 ± 2.2 years. Twenty other subjects received multitasking training, which is not directly related to the purpose of this study (for details, see the ref. 35). One control subject who could not participate in the postexperiment as planned because of the illness was excluded from the study as previously described^35^. A flowchart of this study is shown in Fig. 1.

All subjects had normal vision and no history of neurological or psychiatric illness, which were assessed using a routine questionnaire. Handedness was evaluated using the Edinburgh Handedness Inventory^36^. Written informed consent was obtained from each individual for the projects in which they participated. The Ethics Committee of Tohoku University approved all procedures. All experiments were performed in accordance with the institutional guidelines. Subjects were remunerated based on the extent to which they participated in experiments. For additional details on the subjects, please see Supplemental Methods.

### Procedure

As described in our previous study^13^, the WMT program comprised in-house developed Borland C++ programs of four computerized tasks. Subjects performed approximately four weeks (27 days) of training (approximately 40 min per day on an average). Subjects of the no-intervention group participated in pre- and post-experiments based on the recommendations of our previous review^5^. For full details of training procedures, and reasons for the use of the no-intervention group, see Supplemental Methods. The length of 4–5 weeks of cognitive training is one of the most of widely taken methods and this length is known to cause a wide range of neural plasticity related to cognitive training^13, 37–39^.

### Training tasks

As described in our previous study^13^, four WMT tasks were presented during each training session. In all training tasks, difficulty (number of items to be remembered) was modulated based on performance. The four WMT tasks were as follows: (a) A visuospatial WM task, (b) An auditory backward operation span task, (c) A dual WM task, and (d) a dual N-back task. Full details of these tasks are reproduced in the Supplemental Methods.

### Image acquisition

MRI data were acquired using a 3-T Philips Achieva scanner. As in our previous study^13^, 34 transaxial gradient-echo images (64 × 64 matrix, TR = 2000 ms, TE = 30 ms, flip angle = 70°, FOV = 24 cm, 3.75 mm slice thickness) covering the entire brain were acquired using an echo-planar sequence for rsfMRI analysis. For this scan, 160 functional volumes were obtained while subjects were resting, and during resting state scans, subjects were instructed to stay as motionless as possible with their eyes closed and not to sleep or think about anything in particular, as described previously^40, 41^. Diffusion-weighted data were acquired using a spin-echo EPI sequence, and images with no diffusion weighting (*b* value = 0 s/mm^*2*^ = 0 image) were acquired using previously described methods^42^. After calculation, fractional anisotropy (FA) images and mean diffusivity (MD) maps were obtained, and the resulting images were used exclusively to create templates. These and scans from previous studies^13^ were in most cases taken during the same session.

### Preprocessing and individual-level analysis of imaging data

Preprocessing of imaging data was performed using SPM8 implemented in Matlab and SPM8’s extension software DPARSF (Data Processing Assistant for Resting-state fMRI).

Series of BOLD images for each session and subject were segmented and independently normalized on the basis of modified diffeomorphic anatomical registration using exponentiated lie algebra (DARTEL)-based methods^43^ to give images of 3.75 × 3.75 × 3.75 mm^3^ voxels. Normalized series of BOLD images were processed for individual level analyses using DPARSF. Initially, 26 nuisance covariates included mean signals from voxels within the white matter mask, mean signals from voxels within the CSF mask, and Friston 24 motion parameters. Processed images were spatially smoothed with 8-mm FWHM, and the resulting images were masked with the whole brain mask.

Analyses of fALFF were performed using DPARSF software as previously described^44, 45^, and the sum of amplitudes across 0.01–0.08 Hz were divided by that of the entire frequency range. After preprocessing, fMRI data were temporally band-pass filtered (0.01 < f < 0.08 Hz) to reduce low frequency drift and high frequencies. Weighted DC measures were calculated using DPARSF as previously described^46^.

In addition, regional homogeneity images were calculated according to previously described procedures^47^. In these experiments, spatially normalized rsfMRI images were band-pass filtered (0.01 < f < 0.08 Hz) and were masked by the whole brain mask. Then, regional homogeneity values were calculated to indicate similarities of the time series of a given voxel to its nearest 26 voxels. After normalization, the resulting images were spatially smoothed using 8-mm FWHM. For full details of these procedures, see Supplemental Methods.

### Group-level statistical analyses of imaging data

In the group-level analysis, we compared changes in rsfMRI measures following the intervention period between the WMT and control groups, and by this group difference, we deduced the effects of WMT on these measures. In group-level imaging analyses, we tested for group-wise differences in rsfMRI measurements after WMT and subsequently performed voxel-wise one-way ANCOVAs using Biological Parametrical Mapping^48^ using SPM5 software. Dependent variables included fALFF, DC, or regional homogeneity values from postscans at each voxel, and independent variables included corresponding image values from prescans at each voxel, regional gray matter densities from pre- and postscans at each voxel (to rule out possible effects of changes in regional gray matter structure), volume-level mean framewise displacements of pre- and postscan, age, and sex. For the rationale for the use of ANCOVA instead of repeated-measures ANOVA, please see Supplemental Methods.

Regions of significance were inferred using cluster-level statistics^49^ (meaning, we used default SPM’s cluster size test). Only clusters with *P* < 0.05, after correction for multiple comparisons at cluster size with a voxel-level cluster-determining threshold of *P* < 0.005 uncorrected, were considered statistically significant in this analysis^2, 13^. This voxel level cluster-determining threshold has been used in previous studies^2, 13^.

## Results

### Training data and behavioral data

As described in our previous investigations of the effect of WMT on resting-state FC, regional cerebral blood flow at rest (resting-rCBF), and regional gray matter volume using data from the same subjects^13^, subjects in the WMT group completed an average of 25.87 ± 2.18 sessions and at least 17 sessions during the 27-day intervention period. The best performance of all four trained WM tasks during the last three training sessions was significantly improved compared with that during the first three training sessions (paired t-test, P < 0.001). Details of training data and training-related changes in cognitive test performance scores (such as WM tasks) have been described previously^13^ and are reproduced in Supplemental Table 1.

Volume-level mean framewise displacements of pre-scans^50^ did not differ significantly between groups (WMT, 0.158 ± 0.030; control, 0.152 ± 0.044). After correcting for the effects of volume-level mean framewise displacements of the post scan, ANCOVA revealed no significant differences between groups (WMT, 0.158 ± 0.033; control, 0.167 ± 0.052; P = 0.138, F = 1.211, one-tailed ANCOVA; WMT < control).

### The effect of WMT on DC

**Figure 2:**
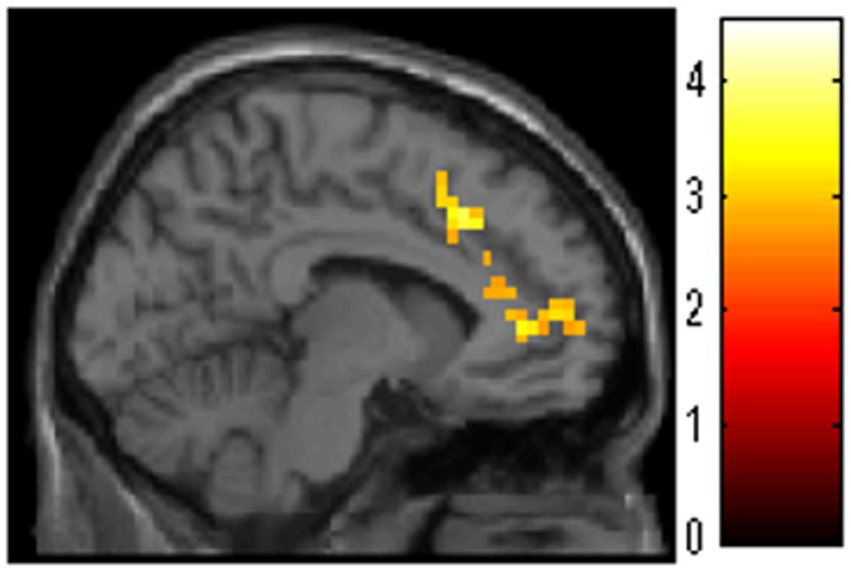
Larger increases in degrees of centrality (DC) were observed in the working memory training (WMT) group compared with the control group. Results are shown with a threshold of P < 0.05 and data were corrected for multiple comparisons at the cluster size with an underlying uncorrected voxel-level of P < 0.005. These data show that compared with no intervention (control), WMT significantly increased DC in the anatomical cluster that spread from the medial prefrontal cortex to ventral and dorsal anterior cingulate cortexes.

After correcting for the effects of confounding variables, whole brain analyses showed significantly greater DC in the anatomical cluster that spread from the medial prefrontal cortex to the ventral and dorsal ACC (Fig. 2) in the WMT group compared with the control group [x, y, z = −11.25, 45, 3.75 t = 4.54, P = 0.005, corrected for multiple comparison (cluster size test) at the whole brain level, 5642 mm^3^, Table 1].

**Table 1:** Brain regions with significant WMT-related changes in rsfMRI measures.

Area		MNI coordinates			*t*-score	Corrected *P* value	Cluster size (mm^3^)
		x	y	z			
WMT-related increase of DC							
Medial prefrontal cortex/Dorsal and Ventral anterior cingulate cortex		−11.25	45	3.75	4.54	0.005^a^	5642
WMT-related increase of fALFF							
Superior frontal gyrus/Frontopolar/Dorsomedial prefrontal cortex		15	45	48.75	4.12	<0.001^a^	9387
WMT-related increase of regional homogeneity							
Precuneus/Posterior cingulate cortex/Posterior parietal cortex		3.75	−52.5	52.5	4.24	0.002^a^	8701
WMT-related decreases in regional homogeneity							
Temporal pole/Middle temporal gyrus/Inferior temporal gyrus	R	56.25	3.75	−22.5	4.28	0.003	8016

^a^Corrected for multiple comparison at the cluster size at the whole brain level.

^b^Corrected for multiple comparison at the cluster size within the region of interest.

### The effect of WMT on fALFF

**Figure 3:**
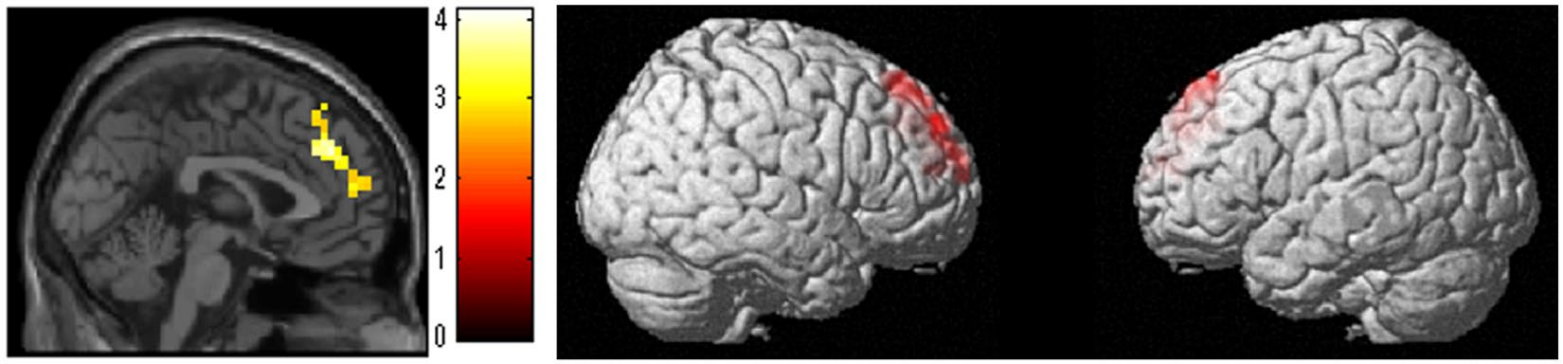
Larger increases in fractional amplitudes of low-frequency fluctuations (fALFF) in the working memory training (WMT) group compared with the control group. Results are shown with a threshold of P < 0.05 and were corrected for multiple comparisons at the cluster size with an underlying uncorrected voxel-level of P < 0.005. These data show that compared with the no intervention (control) group, WMT resulted in significant increases in fALFF in the anatomical cluster that spread mainly from the right superior frontal gyrus, to the dorsomedial prefrontal cortex, the supplementary motor area, and the frontopolar area.

After correcting for confounding variables, whole brain analyses revealed significantly greater increases in fALFF in the anatomical cluster that mainly spread in the right superior frontal gyrus, the frontopolar, and the dorsomedial prefrontal cortex in the WMT group compared with the control group [Fig. 3, x, y, z = 15, 45, 48.75, t = 4.12, P < 0.001, corrected for multiple comparison (cluster size test) at the whole brain level, 9387 mm^3^, Table 1].

### The effect of WMT on regional homogeneity

**Figure 4:**
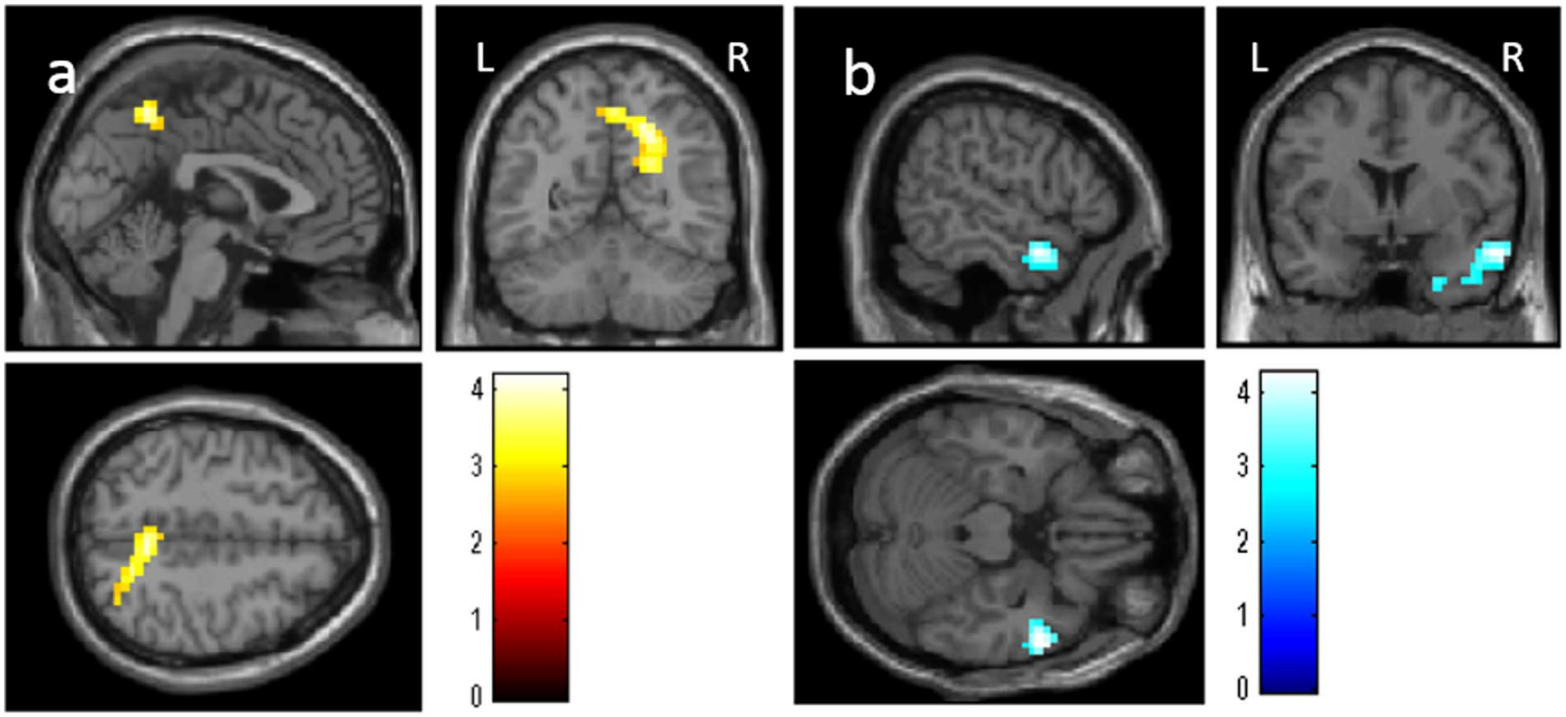
Changes in regional homogeneity in the working memory training (WMT) group compared with the control group. (**a**) Results are shown with a threshold of P < 0.05 and were corrected for multiple comparisons at the cluster size with an underlying uncorrected voxel-level of P < 0.005. The three panels show WMT-related increases in regional homogeneity. (**a**) Increases in regional homogeneity in the WMT group compared with the control group; Compared with the no intervention (control), WMT resulted in significant increases in regional homogeneity in the anatomical cluster that included the precuneus, posterior cingulate cortex, and the right posterior parietal cortex. (**b**) Decreases in regional homogeneity in the WMT group compared with the control group; Compared with the control intervention (no intervention), WMT resulted in a significant decrease in regional homogeneity in the anatomical cluster that included the area around the right temporal pole.

After correcting for confounding variables, whole brain analyses revealed significantly greater increases of regional homogeneity in the anatomical cluster that was spread around the areas of the precuneus, right posterior cingulate cortex, and the right posterior parietal cortex [Fig. 4a, x, y, z = 3.75, −52.5, 52.5, t = 4.24, P = 0.002, corrected for multiple comparison (cluster size test) at the whole brain level, 8701 mm^3^, Table 1) in the WMT group. Moreover, significantly greater decreases in regional homogeneity were identified in the anatomical cluster that spread around the areas of the right temporal pole in the WMT group compared with the control group (Fig. 4b, x, y, z = 56.25, 3.75, −22.5, t = 4.28, P = 0.003, corrected for multiple comparison (cluster size test) at the whole brain level, 8016 mm^3^, Table 1).

## Discussion

To the best of our knowledge, this study is a novel investigation of the effects of WMT on global and local information processing and the strength of spontaneous brain activity through DC, fALFF, and regional homogeneity in healthy young adults. Partly consistent with our hypotheses, WMT increased DC in the anatomical cluster that spread from the dorsal ACC to the ventral ACC and the mPFC. Furthermore, WMT increased fALFF in the anatomical cluster that was mainly spread around the right DLPFC, dorsomedial prefrontal cortex, and frontopolar area, but also included the mPFC area. Finally, WMT increased regional homogeneity in the anatomical cluster that spread from the precuneus to posterior cingulate cortex and posterior parietal cortex, and decreased regional homogeneity in the anatomical cluster that was spread around the right temporal pole. These results cannot be explained by the effects of preexisting group differences in rsfMRI measures, gray matter structural changes, or the effects of head movements.

The present findings extended the previous studies of WMT, rsfMRI, regional cerebral blood flow during rest, and other modalities. In relation to the present findings, our previous study^13^ suggested that WMT leads to increased resting state functional connectivity between key nodes of the DMN and between key nodes of the EAS and extensive areas in the cortex, and to decreased resting state functional connectivity between key nodes of the DMN and EAS. In the present study, we investigated the effects of the recently developed rsfMRI parameters fALFF, DC, and regional homogeneity^51^, which are independent of seed selections.

The present results of DC may suggest that WMT increased the role of the cortical hub of the anterior brain. DC primarily reflects the extent of functional connectivity of the area with the rest of the brain. Therefore, the areas with strong DC are considered as the cortical hub^15^. In addition, the area from the medial prefrontal cortex to the dorsal anterior cingulate is the major brain hub in the anterior brain^15^. The present data may be considered to suggest that WMT strengthens the anterior major cortical hub. While it is known that WM performance is positively correlated with DC in this area^18^, the ensuing implications are unclear because few studies offer appropriate context. Nonetheless, a previous study showed that activity in these regions during resting state correspond with mind wandering^52^. Thus, increased DC in these regions may reflect increased information integration in the entire brain for mind wandering. Other interpretations involve the function of the dorsal ACC, which is related to attention^53^. Hence, the present changes may reflect increased attention toward these extensive areas after WMT. But these are pure speculations and future studies are required to precisely characterize the relevance of DC in these areas.

Using fALFF, which may reflect the magnitude of neural activity^20, 21^, our findings suggested that increased strength of intrinsic brain activity occurs in extensive areas of the dorsal part of the prefrontal cortex. Furthermore, in our previous study^13^ we showed that increases in resting state CBF occurred in the right lateral prefrontal cortex, potentially reflecting increasing numbers of neural components (and resulting metabolic demand), blood vessels, and increases in intrinsic neural activity. Our study also indicates an increase in regional gray matter structure in the extensive areas of the prefrontal cortex and ACC. Thus, using this recently developed fALFF measurement^51^, we successfully advanced the understanding of multiple aspects of neural plasticity in the cortex following WMT.

The implications of the present fALFF findings are unclear because few previous studies report fALFF experiments. However, ALFF and fALFF have been shown to be positively correlated with WM performance in this area^26^. Furthermore, as noted in the Introduction, ALFF has been associated with executive functions in the medial part of the left superior frontal gyrus^54^, and fMRI studies showed that these areas are involved in cognitive manipulation of information retained in the brain (DLPFC)^55^, computation of prediction (possibly when involving others) (ref. 56, and evaluation of internally generated information (rostrolateral prefrontal cortex and frontopolar area)^57^. Thus, increased fALFF may reflect changes in those cognitive activities under default conditions, and the cluster is extensive and involves mPFC areas. Changes in cognitive activities involving the mPFC, such as mind wandering, may also exist. But these are speculations and future studies could investigate these problems using more detailed experimental paradigms involving fALFF.

WMT-induced increases in regional homogeneity between the precuneus and the posterior parietal cortex may reflect increased integrity of relevant information processing in these areas. Regional homogeneity reflects temporal similarities in brain activity compared with adjacent neighbors. Thus, the present results directly suggest increased local information processing in these areas. On the other hand, the posterior parietal cortex is a key neural locus for mental representations of the visual world^58^. Regional homogeneity in this area is also positively correlated with WM performance^34^. The precuneus is an important node in the DMN, as this area has several connections to other areas and integrates both external and self-generated information to produce higher order mental activity^59^. Owing to the use of these information types, this region may be involved in visuospatial imagery, episodic memory retrieval, self-processing operations, and perhaps self-consciousness^59^ and mind wandering^52^. Speculatively, regional homogeneity in these areas may reflect local integrity of information processing, and the present WMT-induced changes likely reflect increases in such integrity. Congruent with this speculation, as opposed to an increases in cognitive function resulting from WMT, a wide range of psychiatric and neurological diseases and cognitive dysfunctions are consistently associated with reduced regional homogeneity in this area^29–32^.

We speculate that WMT-related decreases of the right temporal pole might represent a type of reduced processing of negative emotions, because this area gathers inputs from a wide range of areas and generates complex emotional responses^60^. Higher regional homogeneity in these areas or adjacent areas is associated with longer durations of migraine^61^, and suicide attempts^62^. Furthermore, remission from major depressive disorder with panic disorder is associated with decreased regional homogeneity in these areas^63^. Thus, regional homogeneity in this area may reflect some forms of negative emotional reduction caused by WMT.

In summary, we determined the effects of WMT on global information processing and spontaneous brain activity strength in EAS and DMN. The present data suggest WMT-induced plasticity in the strength of information processing (fALFF), global information processing (DC), and local information processing (regional homogeneity) in the areas of these networks. These measurements reflect previously described alterations in clinical states. Thus, the results that showed these can be changed through short-term cognitive training may provide the basis for new insights into neural plasticity, and may further suggests the possible utility and clinical application of WMT.

## Electronic supplementary material

Supplementary online material
