# Supplementary material for: Neural plasticity in amplitude of low frequency fluctuation, cortical hub construction, regional homogeneity resulting from working memory training
Source: Sci Rep. 2017 May 3;7:1470. doi: 10.1038/s41598-017-01460-6 (PMC5431219; doi:10.1038/s41598-017-01460-6)
Supplement: Supplementary file 1 — Supplementary online material [file 41598_2017_1460_MOESM1_ESM.pdf]

Supplemental online material

Neural plasticity in amplitude of low frequency fluctuation, cortical hub construction, regional homogeneity resulting from working memory training

Hikaru Takeuchi[1], Yasuyuki Taki[123], Rui Nouchi[4], Atsushi Sekiguchi[2,5], Yuka Kotozaki[6], Seishu Nakagawa[5], Carlos Makoto Miyauchi[5], Yuko Sassa[1], Ryuta Kawashima[1,5,6]

[1]*Division of Developmental Cognitive Neuroscience, Institute of Development, Aging and Cancer, Tohoku University, Sendai, Japan*

[2]*Division of Medical Neuroimaging Analysis, Department of Community Medical Supports, Tohoku Medical Megabank Organization, Tohoku University, Sendai, Japan*

[3]*Department of Radiology and Nuclear Medicine, Institute of Development, Aging and Cancer, Tohoku University, Sendai, Japan*

[4]*Human and Social Response Research Division, International Research Institute of Disaster Science, Tohoku University, Sendai, Japan*

[5]*Department of Functional Brain Imaging, Institute of Development, Aging and Cancer, Tohoku University, Sendai, Japan*

[6]*Smart Ageing International Research Center, Institute of Development, Aging and Cancer, Tohoku University, Sendai, Japan*

Short title: Training alters spontaneous activity

Key words: working memory, training, plasticity, fractional amplitude of low frequency fluctuation, degree centrality, regional homogeneity, resting state

Corresponding author:

Hikaru Takeuchi

Division of Developmental Cognitive Neuroscience, Institute of Development, Aging and Cancer, Tohoku University

4 − 1 Seiryo-cho, Aoba-ku, Sendai 980−8575, Japan

Tel/Fax: +81-22-717-7988

E-mail: takehi@idac.tohoku.ac.jp

Supplemental Methods

Procedure

As described in our previous study [1], the WMT program consisted of in-house developed Borland C++ programs comprising four computerized tasks. Subjects participated in approximately four weeks (27 days) of training (approximately 40 min per day). However, the total training time depended on the level and time between trials. Hence, subjects used the program on their personal computers, and were encouraged to perform the WMT tasks every day, in addition to the two weekly training sessions in the laboratory. Details are described in our previous study [1]. Subjects in the no-intervention group participated in pre- and post-experiments based on (a) the recommendation of the previous review [2], (b) the lack of effects in the active control group in our previous study [3], in which sample characteristics and experimental settings were similar to the present study, and (c) the overwhelming number of studies that, in contrast with popular belief, failed to show effects in active control groups [4-6]. Moreover, active control groups are inappropriate controls due to specific effects of active control training on measures where they aren't supposed to have specific effects [2]. The lack of an active control or no control groups combined with within-training group analyses with training

variables are gold standard procedures for imaging studies of cognitive training [2]. In particular, no previous studies show that these well-controlled active interventions affect resting state fMRI measures, and to the best of our knowledge, there is no persuasive scientific theory that suggests changes to resting state fMRI measurements. Hence, inclusion of both active control and no intervention groups is ideal [2], but is usually suboptimal from a statistical perspective [2].

Training tasks

As described in our previous study [1], four WMT tasks were presented during each training session. In all training tasks, difficulties (number of items to be remembered) were modulated based on subjects' performance. The four WMT tasks were as follows: (a) a visuospatial WM task, (b) an auditory backward operation span task, (c) a dual WM task, (d) a dual N-back task.

(a) In the visuospatial WM task, circles are presented one at a time (1/s rate) in an interface of 10 irregularly distributed squares (circles are presented in one of these squares). After the presentation of stimuli, subjects indicate the location and order of the presented stimuli by clicking on a computer screen with a mouse. (b) In an auditory backward operation span task, pairs of single digits (0–9) are verbally presented at a rate

of 1 pair/3 s. Within this 3-s period, one digit is presented per second, but in the final second, no stimuli are presented. Therefore, four pairs of digits are presented as follows: 1, 3, no stimulus; 4, 9, no stimulus; 3, 7, no stimulus; and 2, 5, no stimulus. Subjects were required to remember the value that was the sum of the presented pairs of digits, and the order in which they were presented (in the above example, they were required to remember 4, 3, 0, 7). After presentation, subjects were required to repeat the sequence by pressing numbered buttons on the screen in reverse order (7, 0, 3, 4 in the above example). (c) In the dual WM task, which is similar to that of a previous study [7], subjects concurrently performed a visuospatial WM task and an auditory digit span task. In that study, circles were presented one at a time at a rate of 1/3 s in the same interface used for the task (a). After the presentation of stimuli, subjects indicated the location and order of the presented stimuli by clicking on a computer screen with a mouse. One digit (0–9) was verbally and simultaneously presented with the circle presentation. After the presentation of stimuli, subjects indicated the digits and the order of stimuli by pressing numbered buttons on the screen in the order of presentation. Subjects could perform either task first. (d) In the dual WM task, which was similar to that in a previous study of WMT [8], squares at eight different locations were sequentially presented on a computer screen at a rate of 3/1 s (stimulus length, 500 ms;

inter-stimulus interval, 2,500 ms). Simultaneously, one of eight consonants was sequentially presented using headphones and a response was required whenever one of the presented stimuli matched the stimuli previously presented at the (n) position in the sequence. Additional details regarding the practical aspects of the task procedures and training are described in our previous study [1]. Training times for each task were not necessarily exactly equal among the four tasks, although tasks were performed in the fixed order [(a)–(d)]. The same task was not used for assessment prior to the intervention period.

Preprocessing and individual-level analysis of imaging data

Preprocessing of imaging data was performed using SPM8 implemented in Matlab and SPM8's extension software DPARSF (Data Processing Assistant for Resting-state fMRI). In the following procedures, we avoided co-registration and co-normalization procedures (which use the same normalization parameters for both pre- and post-images) for pre- and post-images, including registration of mean images to pre- and post-images, because of concerns of possible bias or problems occurring when image properties were substantially different between pre- and post-images [9].

For each session and each subject, the first image of a series of BOLD images was skull stripped by masking the images using the threshold of a given signal intensity from

spatially smoothed (using 8 mm FWHM) BOLD images. This skin-skull-stripping procedure was performed so that these parts were not treated as the outer edge of the brain parenchyma in the preprocessing procedures. Furthermore, the skull-stripped BOLD image was co-registered to a previously created custom made skull-stripped EPI template. The series of BOLD images for each session for each subject was slice-timing corrected and realigned using DPARSF. The series of BOLD images for each session for each subject were segmented and independently normalized on the basis of the modified diffeomorphic anatomical registration through exponentiated lie algebra (DARTEL)-based methods [10] to give images with 3.75 × 3.75 × 3.75 mm^3 voxels. In this process, a custom template was also created [10], and the whole brain mask, which comprises voxels that show gray matter tissue probability + white matter tissue probability + cerebrospinal fluid (CSF) tissue probability >0.1, the mask of the areas that are likely to be white matter (white matter tissue probability >0.99, to avoid contamination of signals from white matter), and the mask of the areas that are strongly likely to be CSF (CSF tissue probability >0.99, to avoid the contamination of signals from other tissues) were created from the custom template.

The normalized series of BOLD images were processed by DPARSF for individual level analysis. Initially, 26 nuisance covariates (including mean signals from the voxels within the white matter mask, mean signals from the voxels within the CSF mask, and Friston 24 motion parameters were analyzed. The Friston 24-parameter model (six head motion parameters, six head motion parameters one time point before, and the 12 corresponding squared items) [11] was used to regress out head motion effects. Recent work indicates that regressing out Friston 24-parameters is more effective than other movement correction methods, such as corrections for rigid-body using six

parameters, derivative 12 parameters, and 12 voxel-specific regressors [12]. To further eliminate residual effects of motion on rsfMRI measurements, volume-level mean framewise displacements were computed and used as covariates in second-level analyses [13].

Recent controversy regarding whether or not the whole brain signal should be regressed out in the rsfMRI analysis continues [14], and regressing out the whole brain signal may have merits [12] and weaknesses [15]. In this study, we did not regress out the whole brain signal because it partly reflects global brain activity [16], and it has been suggested that WMT may affect global brain activity [1]. Furthermore, although regressing out the whole brain signal facilitates the distinction between network specific brain activity and global brain activity, our main focuses fALFF and DC do not involve network specific activity and regressing out whole brain activity is particularly conceptually problematic for calculating DC, which is the correlation between one voxel and the whole brain.

Processed images were spatially smoothed with 8-mm FWHM, and the resulting images were masked with the whole brain mask that was created as described above.

Analyses of fALFF were performed using the DPARSF software as previously described [17, 18]. The time series of each voxel was transformed into the frequency domain, and the power spectrum was obtained. Because the power of a given frequency is proportional to the square of the amplitude of that frequency component, the square root was calculated at each frequency of the power spectrum, and the average square

root was then obtained across 0.01–0.08 Hz at each voxel. This average square root was taken as ALFF. For analyses of fALFF, the ratio of the power of each frequency at a low frequency range to that of the entire frequency range (fALFF) was computed as described previously [19, 20]. Specifically, after preprocessing, time series for each voxel were transformed into the frequency domain without band-pass filtering. The square root was calculated at each frequency of the power spectrum, and the sum of the amplitudes across 0.01–0.08 Hz was divided by that of the entire frequency range.

After preprocessing, fMRI data were temporally band-pass filtered ($0.01 < f < 0.08$ Hz) to reduce low frequency drift and high frequency. Furthermore, weighted DC measures were calculated using DPARSF, as previously described [21]. Briefly, Pearson correlation coefficients were initially computed between the time series of all pairs of gray matter voxels, leading to whole-brain functional connectivity matrices for each individual. We calculated this across the whole brain regardless of area because previous studies using large sample sizes have shown the strength of functional connectivity with the periphery of functional networks and gray matter (rather than deep areas within functional areas or gray matter) are important for cognition [10]. Individual correlation matrices were transformed into a Z-score matrix using Fisher's r–z transformation to improve normality. We further computed weighted DC strengths of

voxels as the sum of the connections (Z-values) with all other voxels. As described previously [22], we conservatively restricted our analysis to positive correlations above a threshold of r = 0.25. A relatively higher threshold was chosen to avoid counting voxels with weak correlations that reflect signal noise. Finally, standardized weighted DC maps were acquired by subtracting mean values from within the abovementioned gray matter mask, and then dividing by the standard deviation of the whole gray matter mask [23]. Voxels with higher DC values reflect the sum of the connections for a given voxel and all other voxels, and are thus indicative of their central roles in transferring information across brain regions.

Subsequently, we calculated regional homogeneity images according to previously described procedures [24]. Initially, spatially normalized rsfMRI images were band-pass filtered ($0.01 < f < 0.08$ Hz) and masked using the whole brain mask. A regional homogeneity value was then calculated to measure similarity of the time series of a given voxel with its nearest 26 voxels. After normalization, the resulting images were spatially smoothed using 8-mm FWHM. Normalization of regional homogeneity maps was performed by dividing the regional homogeneity among each voxel by the average regional homogeneity of the whole brain. This regional homogeneity value is known as Kendall’s coefficient concordance (KCC) [25], and is used to measure the

similarity of a time series of a given voxel with that of its nearest voxels in a voxel-wise way, based on the assumption that voxels are temporally similar to their neighbors [26]. Using this method, we measured temporal homogeneity of regional BOLD signals, which reflected the temporal homogeneity of neural activity. The formula to calculate KCC has been expounded in previous studies [27].

Supplemental Table 1.

The average of all subjects' highest performances [the N (number of items to be remembered) in the WM tasks in which subjects answered accurately] on WMT tasks among the first and last three training sessions.

	First three sessions (items)	Last three sessions (items)
Visuospatial WM task	8.82 ± 0.81	11.48 ± 1.92
Auditory backward operation span task	9 ± 1.44	15.18 ± 3.80
Dual WM task	7.58 ± 0.83	10.03 ± 1.38
Dual N-back task	2.82 ± 0.73	5 ± 1.20

Data obtained from 1 subject whose final performance data were missing were removed from the calculation of the average in this task.

References

1. Takeuchi, H. et al. Effects of working memory-training on functional connectivity and cerebral blood flow during rest. *Cortex* **49**, 2106-2125 (2013).
2. Takeuchi, H., Taki, Y. & Kawashima, R. Effects of working memory training on cognitive functions and neural systems. *Rev. Neurosci.* **21**, 427-450 (2010).
3. Takeuchi, H. et al. Working memory training using mental calculation impacts regional gray matter of the frontal and parietal regions. *PLoS ONE* **6**, e23175 (2011).
4. Brown, A.K., Liu-Ambrose, T., Tate, R. & Lord, S.R. The effect of group-based exercise on cognitive performance and mood in seniors residing in intermediate care and self-care retirement facilities: a randomised controlled trial. *Br. J. Sports Med.* **43**, 608-614 (2009).
5. Mahncke, H.W. et al. Memory enhancement in healthy older adults using a brain plasticity-based training program: a randomized, controlled study. *Proc. Natl. Acad. Sci. U. S. A.* **103**, 12523-12528 (2006).
6. Clark, F. et al. Occupational therapy for independent-living older adults. A randomized controlled trial. *JAMA: the journal of the American Medical Association* **278**, 1321-1326 (1997).
7. Dash, D., McNab, F. & Klingberg, T. in 16th Annual Meeting of the Organization for Human Brain MappingBarcelona, Spain; 2010).
8. Jaeggi, S.M., Buschkuehl, M., Jonides, J. & Perrig, W.J. Improving fluid intelligence with training on working memory. *Proc. Natl. Acad. Sci. U. S. A.* **105**, 6829-6833 (2008).

9. Thomas, C. & Baker, C.I. Teaching an adult brain new tricks: a critical review of evidence for training-dependent structural plasticity in humans. *Neuroimage* **73**, 225-236 (2013).
10. Takeuchi, H. et al. Association between resting-state functional connectivity and empathizing/systemizing. *Neuroimage* **99**, 312-322 (2014).
11. Friston, K.J., Williams, S., Howard, R., Frackowiak, R.S. & Turner, R. Movement-related effects in fMRI time-series. *Magn. Reson. Med.* **35**, 346-355 (1996).
12. Chao-Gan, Y. et al. A comprehensive assessment of regional variation in the impact of head micromovements on functional connectomics. *Neuroimage* **76**, 183-201 (2013).
13. Power, J.D., Barnes, K.A., Snyder, A.Z., Schlaggar, B.L. & Petersen, S.E. Spurious but systematic correlations in functional connectivity MRI networks arise from subject motion. *Neuroimage* **59**, 2142-2154 (2012).
14. Takeuchi, H. et al. The association between resting functional connectivity and creativity. *Cereb. Cortex* **22**, 2921-2929 (2012).
15. Murphy, K., Birn, R.M., Handwerker, D.A., Jones, T.B. & Bandettini, P.A. The impact of global signal regression on resting state correlations: are anti-correlated networks introduced? *Neuroimage* **44**, 893-905 (2009).
16. Schölvinck, M.L., Maier, A., Ye, F.Q., Duyn, J.H. & Leopold, D.A. Neural basis of global resting-state fMRI activity. *Proceedings of the National Academy of Sciences* **107**, 10238-10243 (2010).

17. Han, Y. et al. Frequency-dependent changes in the amplitude of low-frequency fluctuations in amnestic mild cognitive impairment: a resting-state fMRI study. *Neuroimage* **55**, 287-295 (2011).
18. Wang, Z. et al. Spatial patterns of intrinsic brain activity in mild cognitive impairment and alzheimer's disease: A resting-state functional MRI study. *Hum. Brain Mapp.* **32**, 1720-1740 (2011).
19. Chao-Gan, Y. & Yu-Feng, Z. DPARSF: a MATLAB toolbox for “pipeline” data analysis of resting-state fMRI. *Frontiers in systems neuroscience* **4**, article 13: 11-17 (2010).
20. Wang, L. et al. Amplitude of Low-Frequency Oscillations in First-Episode, Treatment-Naive Patients with Major Depressive Disorder: A Resting-State Functional MRI Study. *PLoS ONE* **7**, e48658 (2012).
21. Wang, X. et al. Disrupted resting-state functional connectivity in minimally treated chronic schizophrenia. *Schizophr. Res.* **156**, 150-156 (2014).
22. Buckner, R.L. et al. Cortical hubs revealed by intrinsic functional connectivity: mapping, assessment of stability, and relation to Alzheimer's disease. *The Journal of Neuroscience* **29**, 1860-1873 (2009).
23. Yan, C.-G. et al. A comprehensive assessment of regional variation in the impact of head micromovements on functional connectomics. *NeuroImage* **76**, 183-201 (2013).
24. Yao, Z., Wang, L., Lu, Q., Liu, H. & Teng, G. Regional homogeneity in depression and its relationship with separate depressive symptom clusters: a resting-state fMRI study. *J. Affect. Disord.* **115**, 430-438 (2009).

25. Kendall, M. & Gibbons, J.D. Rank correlation methods edward arnold. (Oxford University Press, Oxford, 1990).

26. Tononi, G., McIntosh, A.R., Russell, D.P. & Edelman, G.M. Functional clustering: identifying strongly interactive brain regions in neuroimaging data. *Neuroimage* **7**, 133-149 (1998).

27. Zang, Y., Jiang, T., Lu, Y., He, Y. & Tian, L. Regional homogeneity approach to fMRI data analysis. *Neuroimage* **22**, 394-400 (2004).
